# Exploring the Influence of Material Properties of Epoxy Molding Compound on Wafer Warpage in Fan-Out Wafer-Level Packaging

**DOI:** 10.3390/ma16093482

**Published:** 2023-04-30

**Authors:** Wan-Chun Chuang, Yi Huang, Po-En Chen

**Affiliations:** Department of Mechanical and Electromechanical Engineering, Engineering Technology Research & Promotion Center, National Sun Yat-sen University, Kaohsiung 804, Taiwan; m103020101@student.nsysu.edu.tw (Y.H.); m113020029@student.nsysu.edu.tw (P.-E.C.)

**Keywords:** fan-out wafer-level packaging, warpage, epoxy molding compound, finite element analysis

## Abstract

This study investigated the impact of material properties of epoxy molding compounds on wafer warpage in fan-out wafer-level packaging. As there is currently a lack of comprehensive discussion on the various material property parameters of EMC materials, it is essential to identify the critical influencing factors and quantify the effects of each parameter on wafer warpage. The material properties include Young’s modulus of the epoxy molding compound before and after the glass transition temperature (Tg) range of 25–35 °C (E_L_) and 235–260 °C (E_H_), coefficient of thermal expansion (α1, α2), and the temperature change (∆T) between E_L_ and E_H_. Results show that, within the range of extreme values of material properties, E_L_ and α1 are the critical factors that affect wafer warpage during the decarrier process in fan-out packaging. α1 has a more significant impact on wafer warpage compared with E_L_. E_H_, α2, Tg, and ∆T have little influence on wafer warpage. Additionally, the study identified the optimized material property of the epoxy molding compound that can reduce the maximum wafer warpage in the X and Y directions from initial values of 7.34 mm and 7.189 mm to 0.545 mm and 0.45 mm, respectively, resulting in a reduction of wafer warpage of 92.58% (X direction) and 93.74% (Y direction). Thus, this study proposes an approach for evaluating the impact of material properties of epoxy molding compounds on wafer warpage in fan-out wafer-level packaging. The approach aims to address the issue of excessive wafer warpage due to material variation and to provide criteria for selecting appropriate epoxy molding compounds to enhance process yield in packaging production lines.

## 1. Introduction

With the progress in semiconductor industry technologies, chip processes have become increasingly sophisticated, necessitating corresponding adjustments in packaging technology. Although the current advanced packaging technology has successfully resolved many issues, the package, composed of multiple materials, possesses varying mechanical properties, often resulting in warpage problems caused by mismatched mechanical properties during the manufacturing process, particularly during thermal processing with extreme temperature fluctuations. Warpage issues can lead to abnormal equipment operation, reduced equipment uptime, and even structural detachment, resulting in damage to the package.

**Figure 1:**
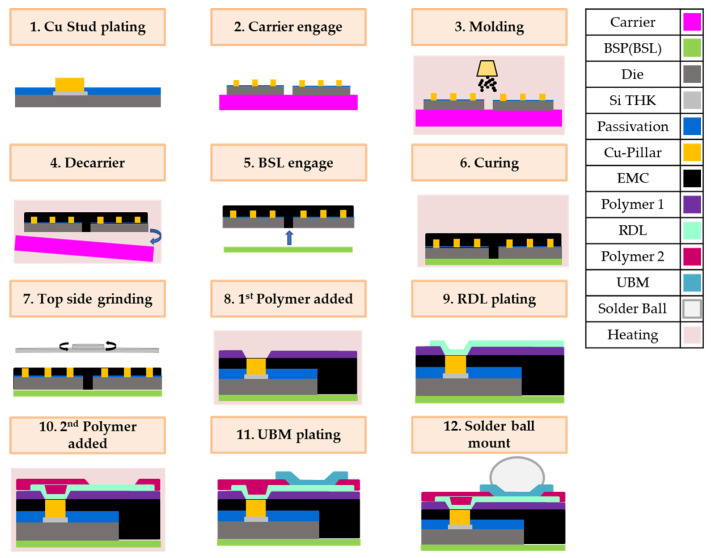
Fan-out process flow.

**Figure 2:**
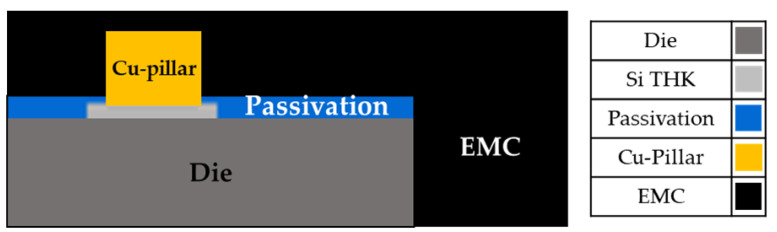
Schematic diagram of a single unit of the decarrier process.

Fan-out wafer-level packaging (FOWLP) is a packaging technology developed to address the demand for high I/O (Input/Output) density. The technology‘s principle involves pulling the required circuitry from the endpoint of the bare die to the redistribution layer (RDL) to form the package. This package does not require a substrate or wire bonding, enabling the package to be thinner. Figure 1 illustrates the process flow of FOWLP. The thermal processes are the 3rd, 4th, 6th, 8th, and 10th processes, and the greatest challenge currently faced by fan-out wafer-level packaging technology is the occurrence of thermal stress in the package during the heating process due to significant temperature variations. This is caused by mismatched coefficients of thermal expansion between different structural layers, which in turn leads to wafer warpage. Based on experimental experience on the production line, the initial warpage will occur during the decarrier process in the 4th process when the material is cooled from 180 °C to 25 °C, and the warpage in this process is the largest among all thermal processes. Therefore, investigating how to reduce the warpage in the 4th process after removing the carrier is one of the important issues of fan-out wafer-level packaging technology. Figure 2 shows a schematic diagram of the structure during the decarrier process, where “Die” represents the chip, “Si THK” represents the stop layer (silicon nitride—a high dielectric constant material), “Passivation” represents the passivation layer, “Cu-pillar” represents the copper pillar, and “epoxy molding compound (EMC)” represents the epoxy molding compound.

Many previous studies [1,2,3,4,5,6,7,8,9,10,11,12,13,14] have focused on evaluating whether changes in the structural design or material selection of the packaging can reduce the amount of wafer warpage caused by the thermal process in fan-out wafer-level packaging (FOWLP). Analyzing the effect of material properties on wafer warpage is one of the important ways of understanding warpage factors and effectively improving wafer warpage. Hou et al. [1] simulated and analyzed the effect of the coefficient of thermal expansion (CTE) of the carrier material on warpage during the encapsulation process and found that minimum warpage occurred when the CTE was reduced from 13.5 ppm/°C to 10.5 ppm/°C, resulting in a 90% reduction in warpage. Lau et al. [2] analyzed the warpage of the packaging structure during post mold cure (PMC) and found that to reduce structure warpage during PMC, the CTE of the glass carrier and the EMC should be as close as possible. Su et al. [3] used shell elements to establish a fan-out panel-level packaging (FOPLP) and studied the effect of three different CTE values of EMC on warpage during the decarrier process. The results showed that reducing the CTE by 0.5 ppm/°C could reduce warpage by 13%. Chiu and Yeh [4] utilized finite element analysis to simulate the thermal process in FOWLP and found that the primary cause of package warpage was the mismatch in the coefficient of thermal expansion (CTE) between different materials and the chemical shrinkage of the EMC. Yang et al. [5] observed that reducing the CTE mismatch between materials on either side of the neutral axis during curing process was beneficial in minimizing warpage. Chen et al. [6] conducted a simulation analysis on the effect of CTE of EMC on warpage after the molding process and found that smaller CTE of EMC resulted in smaller warpage, while an increase in CTE from 7 ppm/°C to 10 ppm/°C led to a 60% increase in warpage. Che et al. [7] analyzed the factors affecting warpage of the wafer packaged using fan-out interposer (FOI) technology. Through simulation, they found that the warpage of the package decreased with decreasing Young’s modulus and CTE of the dielectric layer. In addition, different EMCs also resulted in different warpage trends during the process. Cheng et al. [8] investigated the influence of EMC and carrier material properties on structure warpage during the post-molding cure process of fan-out wafer-level packaging (FOWLP). They found that reducing the CTE of EMC, decreasing Young’s modulus of the carrier, and increasing the CTE of the carrier effectively reduced warpage. Chen et al. [9] analyzed warpage effect of the dry film and the second dielectric film in fan-out wafer-level packaging. The results showed that reducing the elastic modulus or CTE of the dry film and the second dielectric film by 75% could reduce at least 25% of the warpage. Hamaguchi et al. [10] analyzed the impact of Young’s modulus, CTE, and glass transition temperature of EMC on warpage during PMC. The results showed that lower Young’s modulus, CTE, and Tg can effectively reduce warpage, and after optimizing the EMC design, the warpage can be reduced by 65%. Marius et al. [11] analyzed the warpage of two different EMCs during the first thermal cycle in FOWLP and found that different material properties of EMC not only result in different warpages but also cause variations in wafer deformation. Lee et al. [12] found, after analyzing the curing shrinkage of EMC, that the main factor affecting warpage during the curing process is CTE, due to the mismatch between the CTE of EMC and other structural layers, leading to differences in thermal shrinkage and warpage. Wang et al. [13] researched the effect of the chemical shrinkage of EMC on the stress generated in other structures during compression molding. They found that when the chemical shrinkage rate is smaller, the stress generated by the shrinkage deformation of die in EMC will be lower. Chen and Chiang [14] analyzed the warpage shape generated during the decarrier process and found that the asymmetry of the warpage shape is due to the non-uniformity of EMC material properties.

In previous studies [1,2,3,4,5,6,7,8,9,10,11,12,13,14], analysis of the effect of material properties on warpage was mostly focused on the differences in the types of materials or the magnitude of the coefficient of thermal expansion (CTE). However, there are currently few comprehensive discussions on the various material property parameters of the EMC materials, such as Young’s modulus of the EMC before the Tg between 25 °C and 35 °C (E_L_) and after the Tg between 235 °C and 260 °C (E_H_), the thermal expansion coefficient (α1, α2), and the temperature interval (∆T) between E_L_ and E_H_. Table 1 compares the quantification of wafer warpage with respect to EMC parameter characteristics in previous studies [1,2,3,4,5,6,7,8,9,10,11,12,13,14] and this study, as well as the optimization of EMC design characteristics. It is essential to identify the critical influencing factors and quantify the effects of each parameter on the warpage of the structure. Therefore, this study aims to investigate the influence of various material property parameters of EMC on the warpage of FOWLP to develop suitable criteria for evaluating the material properties of the EMC and to improve the process yield in the packaging production line.

## 2. Research Method

To investigate the influence of the material properties of EMC on wafer warpage of fan-out type packaging, this study employed COMSOL Multiphysics software (Burlington, MA, USA) to establish a wafer warpage evaluation model for the decarrier process of fan-out packaging.

### 2.1. Structure Establishment

**Figure 3:**
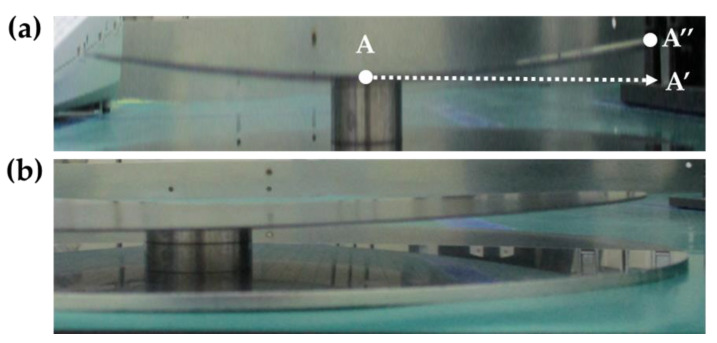
Images of wafer warpage: (**a**) side view; (**b**) magnified view of a specific region.

**Figure 4:**
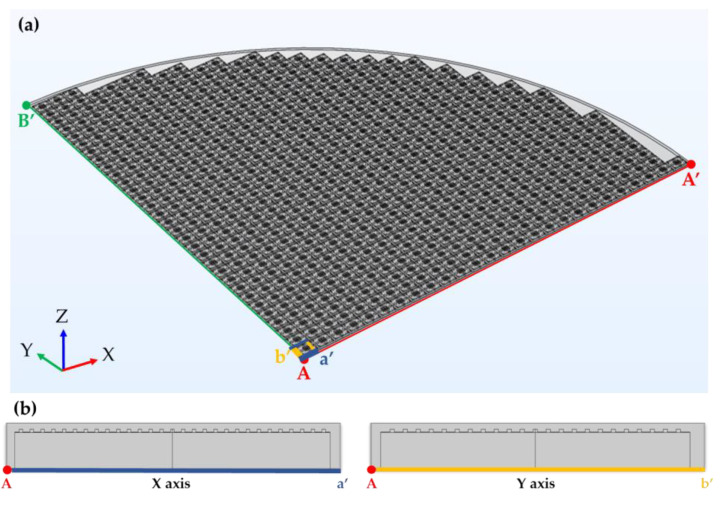
(**a**) One-quarter of the 3D model and (**b**) single unit of the 2D model.

The dimensions of the 12-inch wafer and die-first packaging structure used in FOWLP are shown in Table 2. Maximum warpage was observed at the circumference of the wafer upon cooling the wafer from 180 °C to room temperature (25 °C) during the decarrier process (Figure 3). A 3D 1/4 mapping model was used to analyze the warpage in this study due to the symmetric structure of the wafer. To reduce analysis time, the 3D model was simplified into a 2D model in the X and Y axis cross-sections. Figure 4a shows the 3D 1/4 mapping model of the package and (b) shows a single unit model in the 2D X and Y axis cross-sections of the structure, which include the die, passivation layer, Cu pillar, and EMC.

### 2.2. Boundary Condition Settings

**Figure 5:**
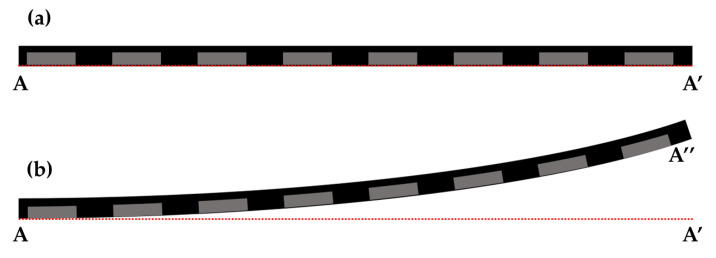
Schematic diagram of the warpage during the decarrier process at (**a**) 180 °C and (**b**) 25 °C.

The process flow of fan-out wafer-level packaging consists of 12 steps (Figure 1) and the generation of wafer warpage is mainly caused by thermal processes during the manufacturing process, resulting in warpage due to the mismatch of mechanical properties of different materials in the structure. Based on practical experience of the production line, maximum wafer warpage in fan-out wafer-level packaging occurs during the decarrier process, when the temperature is decreased from 180 °C to room temperature (25 °C) in 100 s (Figure 5b). As this is the initial step where warpage occurs, the reference temperature for stress-free conditions is set at 180 °C (Figure 5a). Since maximum warpage of the wafer occurs at the circumference, the center position of the model (point A) needs to be fixed, while the region between the center and circumference can deform freely.

### 2.3. Establishing Material Parameters

**Figure 6:**
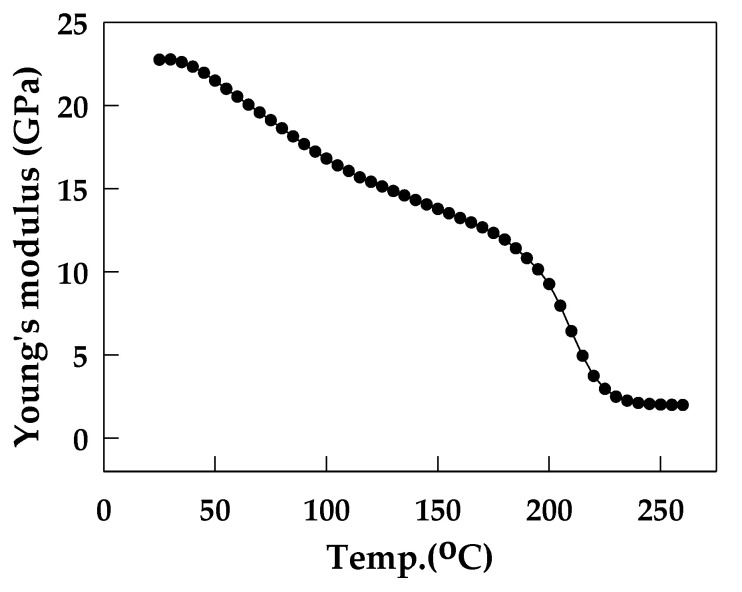
Young’s modulus E(T) of EMC.

During the decarrier process, the structural layer contains die, passivation layer, Cu pillar, and EMC, with the materials being silicon for the die, silicon dioxide for the passivation layer, copper for the Cu pillar, and a polymer material for EMC (Figure 2). The material parameters are listed in Table 3. Since EMC is a polymer material, it exhibits different material properties at different temperatures. Therefore, in this study, Young’s modulus of EMC corresponding to temperature was measured using a dynamic mechanical analyzer (DMA) in a temperature range of 25 °C–260 °C, and Young’s modulus curve (E(T)) is shown in Figure 6. The curve was input into the simulation model to ensure that the material parameters of the model closely matched the real-world situation.

**Figure 7:**
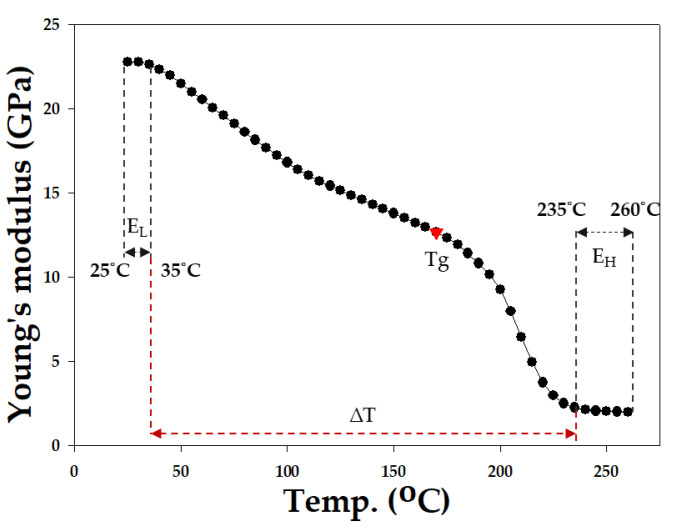
Schematic diagram of the modulation range of Young’s modulus.

In this study, in addition to using the aforementioned EMC, the material properties of EMC were further modified to observe their effects on wafer warpage. Based on a literature review of EMC material parameters [1,2,3,4,5,6,8,15,16], the upper and lower limits of adjustable material values were summarized and organized as shown in Table 4. The range of modulation of Young’s modulus was defined in the study as the temperature interval before and after the Tg point of the EMC, named the temperature interval E_L_ (from 25 °C to 35 °C) and E_H_ (from 235 °C to 260 °C), respectively, with ∆T as the temperature interval between E_L_ and E_H_ (as shown in Figure 7). The thermal expansion coefficients before and after the Tg point were denoted as α1 and α2, respectively. All parameters were adjusted within the ranges specified in Table 4 to analyze the effects of changes in the material properties of the epoxy molding compound on wafer warpage.

## 3. Results

**Figure 8:**
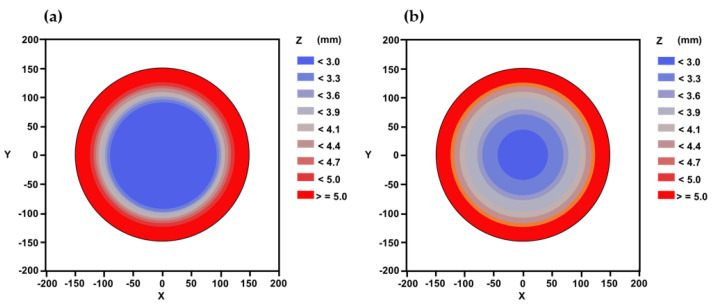
Wafer warpage diagrams of (**a**) simulation and (**b**) experimental results.

Before establishing the criteria for suitable epoxy molding compound material properties, it was necessary to verify the accuracy of the model developed in this study by comparing the results of wafer warpage calculations with experimental results. Table 5 shows the comparison between the calculated wafer warpage values of the model in this study and the experimental values, and Figure 8 presents the comparison between the simulated results and experimental values of the maximum wafer warpage during the decarrier process. Based on the analysis results, the wafer warpage trend is consistent with the experimental results, exhibiting a concave shape, and the error between the simulation and experimental values is only 0.15%. Therefore, the feasibility of the wafer warpage evaluation model for fan-out type packaging established in this study is demonstrated by the results.

## 4. Discussion

### 4.1. Effect of Material Properties on Warpage

Based on experimental observations, it is known that under the decarrier in fan-out type packaging, the wafer will experience significant warpage. Therefore, this study investigated the effects of various material parameters of epoxy molding compounds on the amount of wafer warpage, aiming to identify applicable criteria for reducing wafer warpage. The investigated parameters include: (1) Young’s modulus of epoxy molding compounds (including Young’s modulus (E_L_) in the temperature range of 25 °C–35 °C before the glass transition temperature Tg, and Young’s modulus (E_H_) in the temperature range of 235 °C–260 °C after Tg); (2) The coefficient of thermal expansion (α1, α2); (3) Tg; and (4) The temperature range (∆T) between E_L_ and E_H_.

#### 4.1.1. Effect of Young’s Modulus

**Figure 9:**
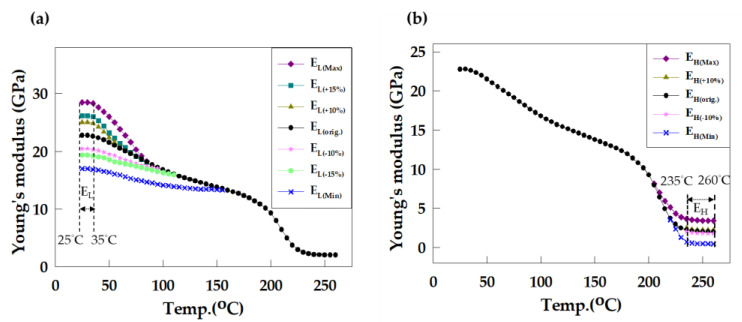
Young’s modulus curves of modulated (**a**) E_L_ and (**b**) E_H_.

**Figure 10:**
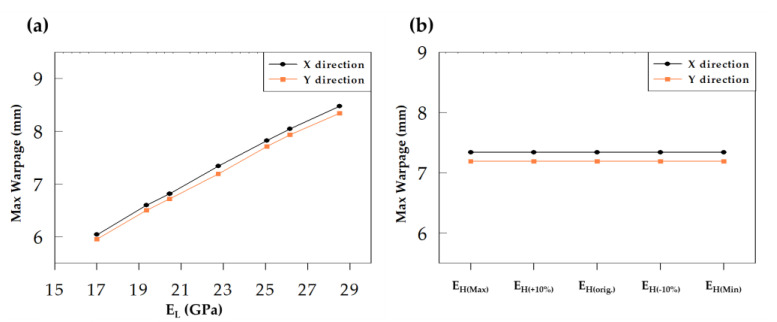
Warpage results of Young’s modulus based on modulating (**a**) E_L_ and (**b**) E_H_.

First, a quantitative analysis of the influence of Young’s modulus parameter of the EMC on wafer warpage was conducted. The range of Young’s modulus modulation of EMC is shown in Table 4. Young’s modulus of E_L_ and E_H_ were then varied positively and negatively by 10%, with E_L(+10%)_ and E_H(+10%)_ representing an increase of 10%, and E_L(−10%)_ and E_H(−10%)_ representing a decrease of 10%. The variation range was set to consider the situation where manufacturers need to fine-tune the characteristics of existing EMCs. The maximum values obtained were named E_L(Max)_ and E_H(Max)_, and the minimum values were named E_L(Min)_ and E_H(Min)_. Young’s modulus curves of the modulated E_L_ and E_H_ are shown in Figure 9. The results of substituting the modulated values into the model are shown in Table 6 and Figure 10. It can be observed that when E_L_ is reduced, wafer warpage is reduced. When E_L_ is reduced by 10%, the maximum wafer warpage in the X direction decreases from 7.34 mm to 6.81 mm, and the maximum wafer warpage in the Y direction decreases from 7.189 mm to 6.717 mm. Compared with the original EMC condition, the maximum wafer warpage in the X direction decreased by about 7.2%, and the maximum wafer warpage in the Y direction decreased by about 6.6%. A decrease of 1% in E_L_ resulted in a reduction of approximately 0.7% in warpage. When E_L_ is modulated to the minimum value, wafer warpage can be further reduced to 6.038 mm (X direction) and 5.953 mm (Y direction), which is a reduction of approximately 17.7% (X direction) and 17.2% (Y direction) (Figure 10a). It can be observed in Figure 10a that there is a linear relationship between the change in E_L_ and the change in wafer warpage, and the smallest warpage was observed at the minimum E_L_ value. On the other hand, modulation of E_H_ had no significant effect on wafer warpage in all the ranges discussed (Figure 10b). Based on these findings, it can be concluded that to reduce wafer warpage by varying Young’s modulus of EMC, product designers should aim to decrease Young’s modulus of EMC prior to the glass transition temperature (Tg).

#### 4.1.2. Effect of CTE

**Figure 11:**
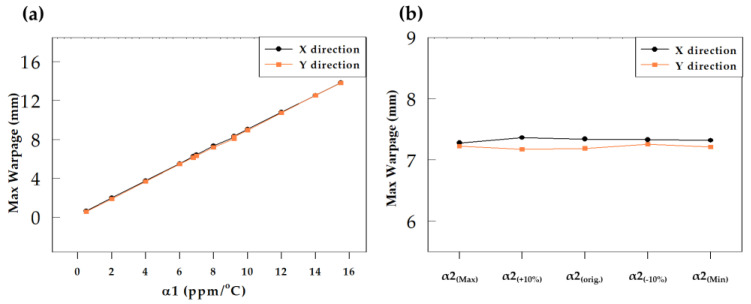
Warpage results of CTE based on modulating (**a**) α1 and (**b**) α2.

Table 4 presents the range of variations in the values of CTE α1 and α2 investigated, and the corresponding wafer warpage values are shown in Table 7. From the results (Figure 11), it can be seen that reducing α1 can effectively reduce wafer warpage. Compared with the original EMC conditions, when α1 decreases by 10%, the maximum wafer warpage in the X direction decreases from 7.34 mm to 6.431 mm (a decrease of 12.4%), and the maximum wafer warpage in the Y direction decreases from 7.189 mm to 6.317 mm (a decrease of 12.1%). A decrease of 1% in α1 leads to a reduction of approximately 1.2% in wafer warpage. Furthermore, when α1 is modulated to its minimum value, the wafer warpage can be significantly reduced to approximately 0.64 mm (X direction) and 0.554 mm (Y direction), a decrease of about 91.3% (X direction) and 92.3% (Y direction). Conversely, increasing α1 increases the wafer warpage (Figure 11a). As shown in Figure 11a, the change in wafer warpage is linearly related to the change in α1, and minimum warpage is achieved when α1 is at its minimum. When α2 is modulated, no significant change in wafer warpage is observed regardless of whether it is increased or decreased (Figure 11b). Comparing the results in Table 6 and Table 7, it can be observed that reducing either Young’s modulus (E_L_) or α1 of the EMC before Tg can improve wafer warpage, but the magnitude of improvement is much greater for α1 than for E_L_. Therefore, the α1 of the EMC is the key factor affecting wafer warpage and designers can significantly reduce wafer warpage by lowering the α1 of the EMC.

#### 4.1.3. Effect of Tg

**Figure 12:**
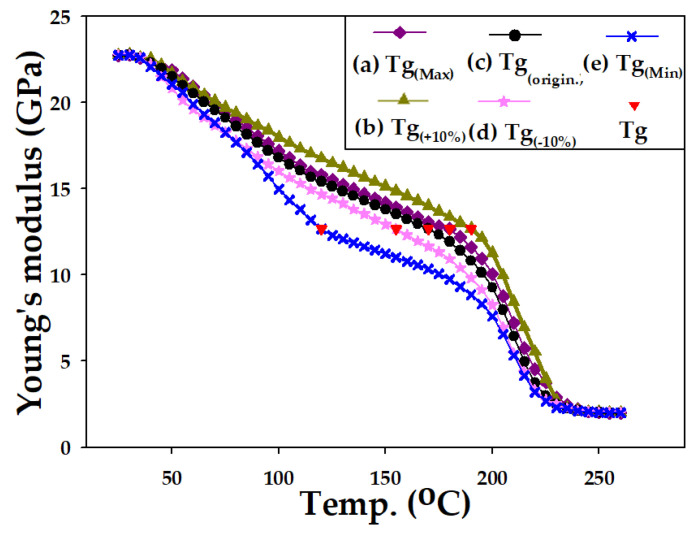
Young’s modulus E(T) for modulated Tg.

**Figure 13:**
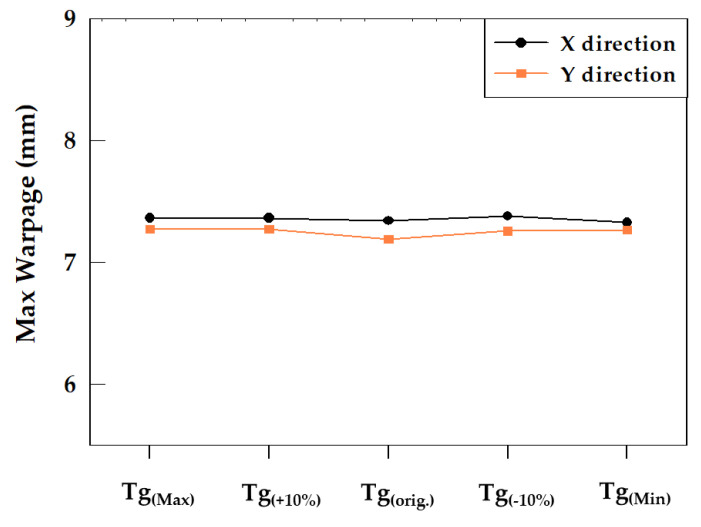
Maximum Warpage for modulated Tg.

The Tg values of EMC and their corresponding wafer warpage discussed in this study are listed in Table 8. Varying the Tg causes slight changes in the values of Young’s modulus curve near Tg. However, since E_L_ is defined as Young’s modulus between 25 °C and 35 °C, and E_H_ is defined as Young’s modulus between 235 °C and 260 °C, there is still a difference in the temperature range between 120 °C and 187 °C, and the Tg point. Therefore, while changing Tg may lead to changes in Young’s modulus near Tg, E_L_ and E_H_ remain unchanged. Young’s modulus curves generated after adjustment are shown in Figure 12. The computational results of the model (Table 8 and Figure 13) show that within all discussed ranges of Tg, the impact on wafer warpage is very slight, with the degree of influence remaining within a 1.2% range of wafer warpage variation.

#### 4.1.4. Effect of ∆T

**Figure 14:**
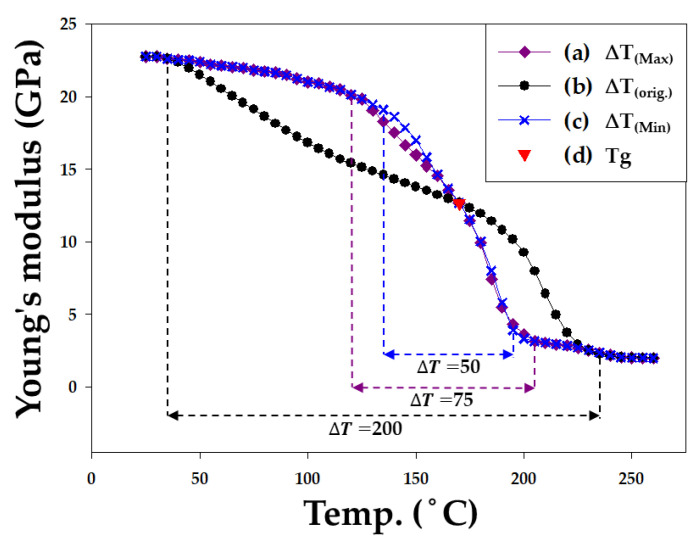
Young’s modulus E(T) of modulated ∆T.

**Figure 15:**
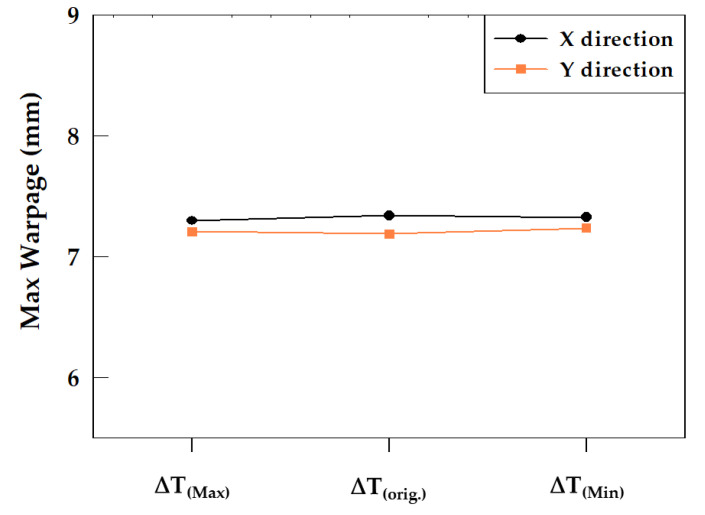
Maximum Warpage of modulated ∆T.

The values of the parameter ∆T and their corresponding wafer warpage are presented in Table 9. Varying ∆T causes significant changes in the values of Young’s modulus curve between E_L_ and E_H_, but E_L_ and E_H_ remain unchanged. Young’s modulus curve after the adjustment is shown in Figure 14. Based on the results (Table 9 and Figure 15), it can be concluded that the variation in ∆T of the EMC has a very minor effect on wafer warpage within the range discussed: the extent of the impact is within 1% of the wafer warpage range.

### 4.2. Optimization Design of EMC Material Properties

Based on the results in Table 6 of Section 4.1.1, it can be observed that a smaller E_L_ value can lead to smaller wafer warpage when varying only one variable in E_L_. The minimum value of E_L_, around −25%, can result in wafer warpage reduction of about −17.74% in the X direction and −17.19% in the Y direction. Additionally, a change of approximately −1% in E_L_ can cause a −0.7% change in wafer warpage and a linear relationship exists between the two parameters until the minimum value of E_L_ is reached. Similarly, based on the results in Table 7 of Section 4.1.2, when varying only α1, a smaller value of α1 leads to a smaller wafer warpage. The minimum value of α1, approximately −90%, can cause a wafer warpage reduction of −91.28% in the X direction and −92.29% in the Y direction, and a linear relationship also exists between the two parameters. Selecting the condition E_L(−10%)_ from Table 6 can cause a −7.22% reduction in wafer warpage in the X direction and a −6.57% reduction in the Y direction. By modulating the same proportion (−10%), selecting the α1_(−10%)_ condition from Table 7 can result in a −12.38% reduction in wafer warpage in the X direction and a −12.13% reduction in the Y direction. Although both E_L_ and α1 parameters decrease by the same magnitude of −10%, changing α1 can improve the degree of wafer warpage reduction more effectively, with a difference of approximately 5% in wafer warpage between the two parameters.

Table 10 presents the results of simultaneously varying two parameters, E_L_ and α1. By comparing the wafer warpage caused by the No. 4 (E_L(+10%)_α1_(−10%)_) and No. 5 (E_L(−10%)_α1_(−10%)_) in Table 10, and the α1_(−10%)_ condition in Table 7, it can be observed that reducing α1 slightly by 10% can cause a −12.38% reduction in wafer warpage in the X direction and a −12.13% reduction in the Y direction. Furthermore, when combined with the variation in the E_L_ parameter by −10% (E_L(−10%)_α1_(−10%)_), the wafer warpage can be further reduced by approximately 5%. Therefore, moderately varying the E_L_ and α1 parameters simultaneously can effectively improve the wafer warpage issue. Among the ranges of the parameters discussed in Table 4, the optimal solution is the No. 9 (E_L(Max)_α1_(Min)_), where the wafer warpage can be reduced from the initial value of 7.34 mm to 0.545 mm in the X direction and from 7.189 mm to 0.45 mm in the Y direction, with a reduction rate of up to 92.58% in the X direction and 93.74% in the Y direction. No. 9 to 14 vary from the maximum to minimum values of E_L_, and a combination with α1_(Min)_ reveals that the reduction in wafer warpage is around 90%, regardless of the E_L_ variation. Therefore, when selecting the optimal EMC material properties, improving α1 should be prioritized.

## 5. Conclusions

This study investigated the impact of material properties of the epoxy molding compound on wafer warpage during the decarrier process in fan-out packaging and identified the key material parameters as Young’s modulus (E_L_) in the temperature range of 25 °C–35 °C before the glass transition temperature (Tg) and coefficient of thermal expansion (α1). The effects of EMC material properties at Tg, E_H_, α2 and the temperature range ∆T between E_L_ and E_H_ on wafer warpage were found to be minor. Furthermore, a model is proposed in this study for quantifying the changes in wafer warpage caused by variations in the critical EMC factors (E_L_, α1), which can provide quantitative assessments for EMC developers to formulate material modification plans. Short-term improvement strategies could involve slight changes to material properties such as adjusting E_L_ and α1 in the same proportion (−10%) to reduce wafer warpage by about 17%. However, to achieve significant improvements, substantial changes to EMC material properties are required under optimized design conditions. Therefore, using the model proposed in this study, it is possible to estimate the effects of changing the material properties of the epoxy molding compound on alleviating wafer warpage in fan-out wafer-level packaging. The proposed optimization design suggestions for EMC material properties can reduce the development cycle for EMC applications, accelerate product development, and improve process yield on packaging lines.

## Figures and Tables

**Table 1:** Quantification of the differences in wafer warpage with respect to EMC parameter characteristics in previous studies [1,2,3,4,5,6,7,8,9,10,11,12,13,14] and this study.

Items	E(E_L_, E_H_)	CTE(α1, α2)	Tg	∆T	Quantified Analysis of Wafer Warpage	Optimization of EMC
[1]	X	α: O	X	X	CTE: O	X
[2]	X	X	X	X	X	X
[3]	X	α: O	X	X	CTE: O	X
[4]	X	X	X	X	X	X
[5]	X	X	X	X	X	X
[6]	X	α: O	X	X	CTE: O	X
[7]	E: O	α: O	X	X	E, CTE: O	X
[8]	E: O	α: O	X	X	E, CTE: O	X
[9]	E: O	α: O	X	X	E, CTE: O	X
[10]	E: O	α: O	O	X	E, CTE, Tg: O	O (warpage: 65%↓)
[11]	X	X	X	X	X	X
[12]	X	X	X	X	X	X
[13]	X	X	X	X	X	X
[14]	X	X	X	X	X	X
This work	E_L_: O, E_H_: O	α1: O, α2: O	O	O	(E_L_, E_H_), (α1, α2), Tg, ∆T: O	O (warpage: 92.58%↓ (X-direction) and 93.74%↓ (Y-direction))

**Table 2:** Specifications of the fan-out wafer-level package.

Information	Value
Wafer size (mm^2^)	150 × 150 × π
Package size (mm^2^)	5.46 × 4.49
Molding compound thickness (µm)	625
Die size (mm^2^)	5.25 × 4.28
Die thickness (µm)	500
Passivation thickness (µm)	6
Cu-pillar pitch (µm)	120

**Table 3:** Material parameters of Cu pillar, die, passivation, and EMC.

	Cu Pillar (Cu)	Die (Si 100)	Passivation	EMC
Young’s modulus (Gpa)	120	131	66	Figure 6
Poisson’s ratio	0.34	0.27	0.17	0.3
Density (kg/m^3^)	8960	2330	2270	2040
CTE (ppm/°C)	16.5	2.8	0.56	α1: 8/α2: 25

**Table 4:** The range of modulation for EMC.

Parameter	Value
Young’s modulus (Gpa)	E_L_: 8.96~28.5/E_H_: 0.45~3.4
Tg (°C)	120~180
∆T (°C)	50~75
CTE (ppm/°C)	α1:0.32~16/α2:22~56

**Table 5:** Comparison of simulation and experimental values of maximum wafer warpage.

Process Step	Max Wafer Warpage (mm)		
	Experimental Value	Simulation Value	Error (%)
4. Decarrier	7.329 ± 0.3 (concave)	X axis: 7.34 Y axis: 7.189	0.15

**Table 6:** Modulated E with the corresponding warpage.

**Modulate** **d** **E_L_**	**Average E_L_** **(Gpa)**	**Max Warpage (mm)**		**Variation (%)**	
		**X Direction**	**Y Direction**	**X Direction**	**Y Direction**
E_L(+25%(Max))_	28.50	8.477	8.342	+15.50%	+16.03%
E_L(+15%)_	26.15	8.046	7.931	+9.62%	+10.32%
E_L(+10%)_	25.05	7.825	7.71	+6.61%	+7.25%
E_L(orig_._)_	22.75	7.34	7.189	0%	0%
E_L(−10%)_	20.45	6.81	6.717	−7.22%	−6.57%
E_L(−15%)_	19.35	6.595	6.504	−10.15%	−9.53%
E_L(−25%(Min))_	17.00	6.038	5.953	−17.74%	−17.19%
**Modulate** **d E_H_**	**Average E_H_** ** (Gpa)**	**Max Warpage (mm)**		**Variation (%)**	
		**X Direction**	**Y Direction**	**X Direction**	**Y Direction**
E_H(Max)_	3.40	7.34	7.189	0%	0%
E_H(+10%)_	2.19	7.34	7.189	0%	0%
E_H(orig_._)_	1.99	7.34	7.189	0%	0%
E_H(−10%)_	1.79	7.34	7.189	0%	0%
E_H(Min)_	0.45	7.34	7.189	0%	0%

**Table 7:** Modulated CTE with the corresponding warpage.

**Modulated α1**	**CTE Value (ppm/°C)**	**Max Warpage (mm)**		**Variation (%)**	
		**X Direction**	**Y Direction**	**X Direction**	**Y Direction**
α1_(+90%(Max))_	α1: 15.5/α2: 25	13.839	13.804	+88.54%	+92.01%
α1_(+75%)_	α1: 14/α2: 25	12.538	12.532	+70.82%	+74.32%
α1_(+50%)_	α1: 12/α2: 25	10.813	10.734	+47.32%	+49.31%
α1_(+25%)_	α1: 10/α2: 25	9.058	8.962	+23.41%	+24.66%
α1_(+15%)_	α1: 9.2/α2: 25	8.337	8.266	+13.58%	+14.98%
α1_(+10%)_	α1: 9/α2: 25	8.223	8.082	+12.03%	+12.42%
α1_(orig_._)_	α1: 8/α2: 25	7.34	7.189	0%	0%
α1_(−10%)_	α1: 7/α2: 25	6.431	6.317	−12.38%	−12.13%
α1_(−15%)_	α1: 6.8/α2: 25	6.272	6.133	−14.55%	−14.69%
α1_(−25%)_	α1: 6/α2: 25	5.516	5.462	−24.85%	−24.02%
α1_(−50%)_	α1: 4/α2: 25	3.753	3.665	−48.87%	−49.02%
α1_(−75%)_	α1: 2/α2: 25	1.984	1.886	−72.97%	−73.77%
α1_(−90%(Min))_	α1: 0.5/α2: 25	0.64	0.554	−91.28%	−92.29%
**Modulated α2**	**CTE Value(ppm/°C)**	**Max Warpage (mm)**		**Variation (%)**	
		**X Direction**	**Y Direction**	**X Direction**	**Y Direction**
α2_(Max)_	α1: 8/α2: 55	7.278	7.223	−0.85%	+0.47%
α2_(+10%)_	α1: 8/α2: 27	7.365	7.175	+0.34%	−0.20%
α2_(orig_._)_	α1: 8/α2: 25	7.34	7.189	0%	0%
α2_(−10%)_	α1: 8/α2: 23	7.329	7.256	−0.15%	+0.93%
α2_(Min)_	α1: 8/α2: 22	7.318	7.212	−0.30%	+0.32%

**Table 8:** Modulated Tg with the corresponding warpage.

Parameter	Tg_(Max)_	Tg_(+10%)_	Tg_(orig_._)_	Tg_(−10%)_	Tg_(Min)_
Young’s modulus (Gpa)	Figure 12-(a)	Figure 12-(b)	Figure 12-(c)	Figure 12-(d)	Figure 12-(e)
Tg (°C)	180	187	170	153	120
CTE (ppm/°C)	α1: 8/α2: 25	α1: 8/α2: 25	α1: 8/α2: 25	α1: 8/α2: 25	α1: 8/α2: 25
Maximum Warpage in X direction (mm)	7.361	7.361	7.34	7.376	7.324
Maximum Warpage in Y direction (mm)	7.271	7.269	7.189	7.255	7.264

**Table 9:** Modulated ∆T with the corresponding warpage.

Parameter	∆T_(Max)_	∆T_(orig_._)_	∆T_(Min)_
Young’s modulus (GPa)	Figure 14-(a)	Figure 14-(b)	Figure 14-(c)
Tg (°C)	170	170	170
∆T (°C)	75	200	50
CTE (ppm/°C)	α1: 8/α2: 25	α1: 8/α2: 25	α1: 8/α2: 25
Maximum Warpage in X direction (mm)	7.299	7.34	7.326
Maximum Warpage in Y direction (mm)	7.208	7.189	7.238

**Table 10:** Modulated E_L_ and α1 with corresponding warpage.

No.	Modulated E_L_ & α1	Max Warpage (mm)		Variation (%)	
		X Direction	Y Direction	X Direction	Y Direction
**1**	E_L(orig_._)_α1_(orig_._)_	7.34	7.189	0%	0%
**2**	E_L(+25%(Max))_α1_(+90%(Max))_	16.302	16.13	+122.10%	+124.37%
**3**	E_L(−10%)_α1_(+10%)_	7.644	7.524	+4.14%	+4.66%
**4**	E_L(+10%)_α1_(−10%)_	6.876	6.758	−6.32%	−6.00%
**5**	E_L(−10%)_α1_(−10%)_	6.05	5.963	−17.58%	−17.05%
**6**	E_L(+25%(Max))_α1_(**orig**_**.**_)_	8.477	8.342	+15.50%	+16.03%
**7**	E_L(+25%(Max))_α1_(**−10%**)_	7.449	7.263	+1.49%	+1.03%
**8**	E_L(+25%(Max))_α1_(**−1**_**_5__%_**_)_	7.238	7.08	−1.39%	−1.52%
**9**	E_L(Max)_α1_(−90%(Min))_	0.545	0.45	−92.58%	−93.74%
**10**	E_L(+15%)_α1_(−90%(Min))_	0.588	0.495	−91.99%	−93.11%
**11**	E_L+10%)_α1_(−90%(Min))_	0.608	0.514	−91.72%	−92.85%
**12**	E_L(−10%)_α1_(−90%(Min))_	0.678	0.59	−90.76%	−91.79%
**13**	E_L(−15%)_α1_(−90%(Min))_	0.692	0.606	−90.57%	−91.57%
**14**	E_L(−25%(Min))_α1_(−90%(Min))_	0.719	0.634	−90.2%	−91.2%

## Data Availability

Not applicable.
